# κ Opioid Receptor-Dynorphin Signaling in the Central Amygdala Regulates Conditioned Threat Discrimination and Anxiety

**DOI:** 10.1523/ENEURO.0370-20.2020

**Published:** 2021-01-12

**Authors:** Madison A. Baird, TingTing Y. Hsu, Rachel Wang, Barbara Juarez, Larry S. Zweifel

**Affiliations:** 1 Department of Pharmacology, University of Washington, Seattle, WA 98115; 2 Department of Psychiatry and Behavioral Sciences, University of Washington, Seattle, WA 98115

**Keywords:** anxiety, central amygdala, dynorphin, fear, κ, opioid

## Abstract

Neuropeptides within the central nucleus of the amygdala (CeA) potently modulate neuronal excitability and have been shown to regulate conditioned threat discrimination and anxiety. Here, we investigated the role of κ opioid receptor (KOR) and its endogenous ligand dynorphin in the CeA for regulation of conditioned threat discrimination and anxiety-like behavior in mice. We demonstrate that reduced KOR expression through genetic inactivation of the KOR encoding gene, *Oprk1*, in the CeA results in increased anxiety-like behavior and impaired conditioned threat discrimination. In contrast, reduction of dynorphin through genetic inactivation of the dynorphin encoding gene, *Pdyn*, in the CeA has no effect on anxiety or conditioned threat discrimination. However, inactivation of *Pdyn* from multiple sources, intrinsic and extrinsic to the CeA phenocopies *Oprk1* inactivation. These findings suggest that dynorphin inputs to the CeA signal through KOR to promote threat discrimination and dampen anxiety.

## Significance Statement

The regulation of fear and anxiety-related behavior are critically dependent on the central nucleus of the amygdala (CeA) region of the brain. Here, we demonstrate that signaling through the κ opioid receptor (KOR) in the CeA regulates conditioned threat discrimination and anxiety related behavior mice. These findings have broad implications for the neural mechanisms that regulate conditioned threat and anxiety-related behavior.

## Introduction

Anxiety-related disorders, including phobias, obsessive compulsive disorders, generalized anxiety, and posttraumatic stress disorder (PTSD) display heterogeneous symptoms. Many of these disorders are based in aberrant information processing of fear-inducing stimuli, resulting in inappropriate responses to perceived threats as well as the sustained state of apprehension known as anxiety (Davis et al., 2010). Learning to discriminate between predictive and non-predictive threat stimuli is adaptive; however, non-discriminative, or generalized threat responding is often maladaptive and is a hallmark of many fear-related disorders (Dunsmoor and Paz, 2015). In experimental systems, such as rodents, conditioned threat discrimination is assessed by pairing an unconditioned stimulus (US) with a conditioned stimulus (CS+). Comparing conditioned responses evoked by the CS+ with responses evoked by an unpaired stimulus (CS–) provides a metric of threat discrimination (Zweifel, 2019).

The amygdala is a key brain region for regulating affective behavior and emotional learning (LeDoux, 2000). The extended amygdala which includes the central nucleus of the amygdala (CeA), bed nucleus of the stria terminalis (BNST), and nucleus accumbens (NAc) shell is also critically involved regulating affective behavioral responses (Fox et al., 2015). The CeA is a heterogeneous and interconnected structure that has emerged as an important central regulator for the acquisition, consolidation, and expression of conditioned threat (Ciocchi et al., 2010; Haubensak et al., 2010; Li et al., 2013) and anxiety-related behavior (Tye et al., 2011; Botta et al., 2015). Anatomically, the CeA is divided into three subregions, the capsular (CeC), lateral (CeL), and medial (CeM) subdivisions; which have divergent inputs, outputs, and roles in fear learning (Duvarci and Pare, 2014). Critical plasticity and local reciprocal inhibition occur during fear learning in the CeL, which inhibits CeM output neurons mediating freezing and defensive behaviors (Ciocchi et al., 2010; Haubensak et al., 2010; Li et al., 2013). Additionally, the CeC receives dense inputs from the lateral parabrachial nucleus (LPN) signaling the US information to neurons expressing PKCδ and Calcrl (Dong et al., 2010; Han et al., 2015; Kim et al., 2017). Thus, the CeA acts as a hub relaying information related to threat discrimination by receiving cue-related signals from extra-amygdalar regions such as sensory cortices and the thalamus (Bienkowski and Rinaman, 2013; Duvarci and Pare, 2014), as well as from the lateral and basolateral amygdala (Tovote et al., 2015), and direct nociceptive information from the spinal parabrachial tract (Han et al., 2015).

Neuropeptides, including corticotropin releasing factor (CRF), dynorphin, enkephalin, tachykin 2, and neurotensin are highly enriched in the CeA (Kim et al., 2017; McCullough et al., 2018), where they have been shown to modulate multiple aspects of conditioned threat and anxiety-related behaviors (Regev et al., 2012; Andero et al., 2014; Fadok et al., 2017; Sanford et al., 2017; Ahrens et al., 2018; Dedic et al., 2018b; Jo et al., 2020). CRF and dynorphin are broadly linked to stress, fear, and anxiety (Land et al., 2008; Bruchas et al., 2009; Chavkin, 2013; Fadok et al., 2017; Sanford et al., 2017; Ahrens et al., 2018; Dedic et al., 2018a; Jo et al., 2020). While CRF is a direct mediator of the stress response, dynorphin is proposed to mediate the dysphoric effects of stress (Land et al., 2008). Broad activation of the dynorphin receptor, κ opioid receptor (KOR), increases depression-like and anxiety-like behavior, and proaddictive behaviors (Shippenberg et al., 2001; Land et al., 2008; Bruchas et al., 2009; Chavkin, 2013). Dynorphin also mediates a variety of adaptive responses to stress, such as analgesia and aversion which increases the motivation to escape from a threat (Knoll and Carlezon, 2010).

Within the CeA, dynorphin is known to increase after acute stress (Chung et al., 2014; Xie et al., 2017; Nation et al., 2018) or local application of CRF (Lam and Gianoulakis, 2011). Dynorphin expression is also increased in CeA somatostatin (SOM)-expressing neurons in a mouse model of anxiety (Ahrens et al., 2018). Suppression of *Pdyn* expression in CRF neurons of the CeA reduces conditioned threat responses (Pomrenze et al., 2019) and pharmacological inhibition of KOR in the CeA disrupts fear-potentiated startle (Knoll et al., 2011); however, the role of KOR-dynorphin signaling in the CeA for threat discrimination remains to be addressed.

To examine the role of CeA KOR and dynorphin in discriminative fear learning and anxiety-like behavior, we reduced *Oprk1* and *Pdyn* expression in the CeA by injecting floxed *Oprk1* and floxed *Pdyn* mice with and adeno-associated virus (AAV) containing an expression cassette for Cre-EGFP (AAV1-Cre-EGFP). Somewhat surprisingly, we found that local CeA KOR, but not dynorphin, is necessary for maintaining normal baseline anxiety-like behavior in an elevated-plus maze (EPM) and for promoting conditioned threat discrimination. In contrast to reducing local dynorphin within the CeA, we found that reducing *Pdyn* expression from local and distal sources to the CeA using a retrograde viral delivery of Cre resulted in increased anxiety-like behavior and conditioned threat generalization. These findings suggest that normal anxiety and discriminative fear learning through KOR signaling in the CeA is likely dependent on distal sources of dynorphin.

## Materials and Methods

### Mice

Male and female *Pdyn^Cre/+^*, *Oprk^Cre/+^*, *Pdyn^lox/lox^*, *Oprk^lox/lox^*mice on a C57BL/6 background were group-housed on a 12/12 h light/dark cycle with free access to food and water. Behavioral experiments were conducted during the light cycle (lights on at 7 A.M.). All procedures were approved by the University of Washington Institutional Care and Use Committee. Males and females were distributed evenly between groups.

### Virus production and surgery

AAV1-FLEX-Synaptophysin-GFP, AAV1-Cre-GFP, AAV1-FLEX-mCherry, AAV1-FLEX-EGFP, CAV2-Cre, and CAV2-FLEX-ZsGreen were produced in house (titer ∼3 × 1012 particles/μl) as described previously (Gore et al., 2013). AAV2retro-FLEX-EYFP was produced by the Washington University Hope Center and was the gift of Michael Bruchas. Viral vectors were stored at −80°C before surgery, and kept on ice on surgery day. Viral vectors were stereotaxically injected (0.5 μl) at a rate of 0.25 μl/min bilaterally into the CeA of isofluorane-anesthetized mice (8–16 weeks old) using the following coordinates: −1.165 mm posterior, ±2.91 mm lateral, and either −4.6 or −4.7 ventral to bregma [according to the Paxinos’ atlas (Paxinos and Franklin, 2001), average injection site was observed at −1.4 mm posterior of bregma]. Controls for local knock-out (KO) were lox/lox injected with AAV1-FLEX-mCherry, wild-type animals injected with AAV1-Cre-GFP, and wild types injected with AAV1-FLEX-mCherry. Controls for retrograde CAV2-Cre mediated KO were wild-type animals injected with CAV2-FLEX-ZsGreen. All mice recovered from surgery for two to three weeks and experiments were run with 10- to 19-week-old mice. All animals used for behavior underwent stringent histology checks to confirm that the virus displayed a bilateral CeA hit without targeting surrounding regions such as the basolateral amygdala or the caudate putamen. Extra-CeA targeting or low levels of viral expression in the CeA usually resulted in 30–50% of mutants’ data being removed from experiments.

### Anxiety: elevated-plus maze (EPM)

EPM experiments were performed on a Med Associates EPM for mice (product #ENV-560A) which has two “closed arms” with black walls, two “open arms” with 1-cm ledges, and white flooring; 10-min sessions begin as the mouse is placed in a closed arm of the arena, with its nose at the edge of the center area boundary. Sessions were recorded from above and mouse center point movement tracked by Ethovision (Noldus) tracking software. Total distance traveled, time spent in open and closed arms, latency to enter the open arm, and frequency of open arm entries were analyzed. Frequency of open arm entries was highly variable and deemed unreliable, as the center point of animals standing at the edge of open arm often togged between the open arm and center area, creating an artificially high number of open arm entries. No differences were found in distance traveled (data not shown) or latency to enter open arm (Extended Data Figs. 1-1, 2-1, 4-1), but anxiety phenotypes could be observed through changes in open and closed arm time; 1-(open arm time/closed arm time) was calculated, with a score of 1 showing maximal anxiety-like behavior that can be detected by this paradigm.

**Figure 1.**
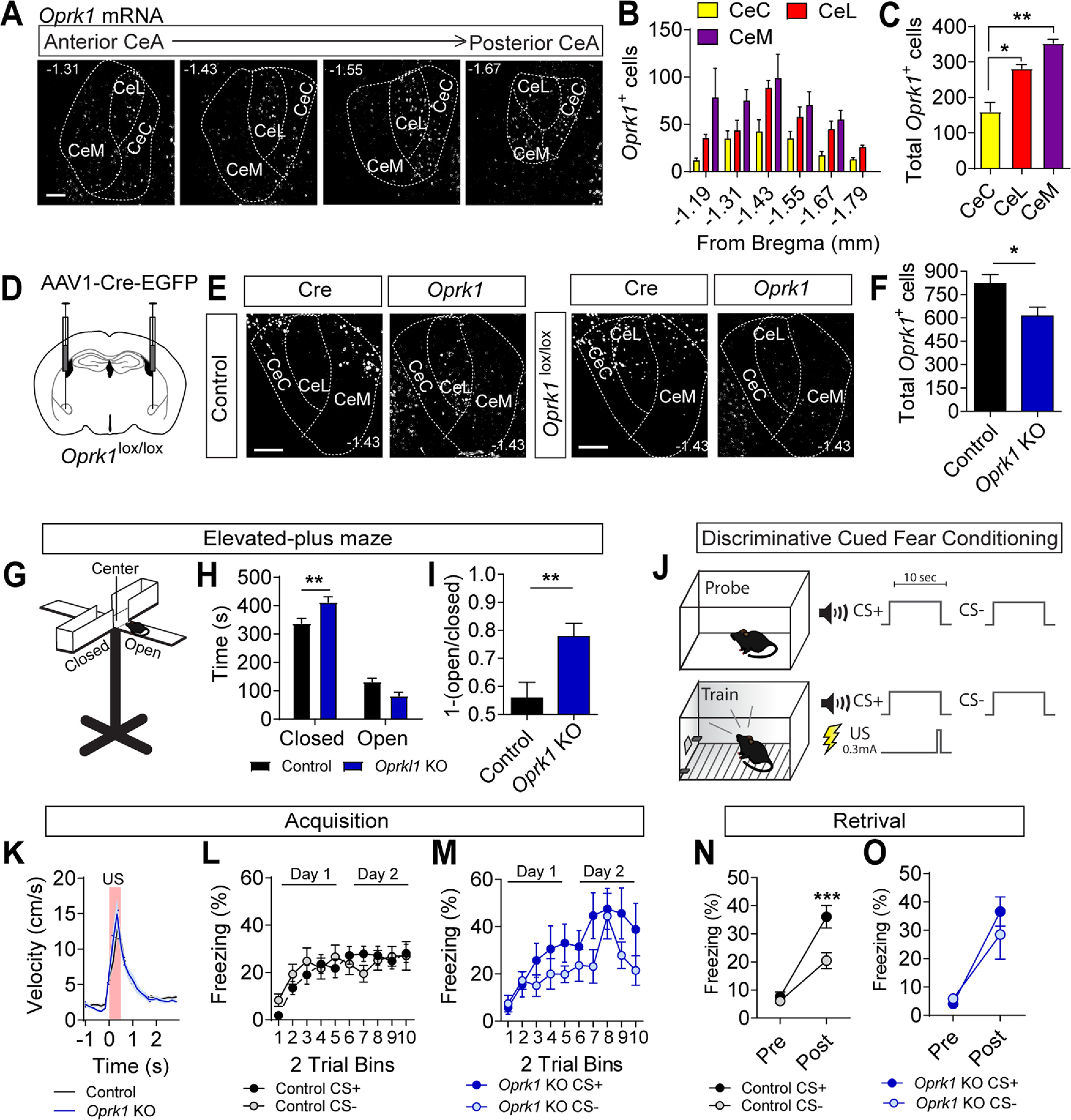
CeA *Oprk1* expression and regulation of anxiety-like behavior and threat. ***A***, Representative images of *Oprk1* mRNA expression along the rostral-caudal extent of the CeA. Scale bar: 100 μm. ***B***, Distribution of cells along the rostral-caudal extent of the CeA-expressing *Oprk1* (*N* = 5 mice). ***C***, Distribution of cells expressing *Oprk1* within the subdivisions of the CeA (one-way ANOVA, *F*_(2,12)_ = 30.23, *p* = 0.01, followed by Tukey’s *post hoc* comparison test, *p < 0.05, **p < 0.01). ***D***, Schematic of injection for AAV1-Cre-GFP into the CeA of *Oprk1^lox/lox^* mice. ***E***, *Oprk1* mRNA in the CeA of control (*Oprk1^+/+^*) and *Oprk1^lox/lox^* mice. Scale bar: 100 μm. ***F***, Quantification of *Oprk1* mRNA-positive cells in the CeA following Cre expression in control (*N* = 5) and *Oprk1^lox/lox^* (*N* = 4) mice (Student’s *t* test, *p < 0.05). ***G***, Cartoon of EPM consisting of open arms, closed arms, and a center transition zone. ***H***, Time spent in open and closed arms of the EPM in control (*N* = 24) and *Oprk1* KO mice (*N* = 10), two-way ANOVA, *F*_(1,58)_ = 13.12, *p* = 0.0006, followed by Bonferroni’s *post hoc* comparisons, **p < 0.01). ***I***, [1-(open/closed arm time)] in control and *Oprk1* KO mice (Student’s *t* test, *p < 0.05). ***J***, Cartoon of discriminatory threat conditioning paradigm. Mice are probed for freezing in response to a CS+ and CS– tone in context A (probe) before conditioning in context B (train). Following training, mice are again tested in context A for conditioned threat responses to the CS+ and CS– tones. ***K***, Average US responses in control (*N* = 24) and *Oprk1* KO mice (*N* = 10) during training. ***L***, ***M***, Average CS+ and CS– freezing in control (***L***) and *Oprk1* KO mice (***M***) during acquisition (control, *N* = 24; *Oprk1* KO, *N* = 10). ***N***, ***O***, Average freezing to CS+ and CS– during probe trials for retrieval in control (***N***) and *Oprk1* KO mice (***O***). Control but not *Oprk1* KO mice showed significant cue discrimination during probe trials (two-way ANOVA, *F*_(1,40)_ = 7.79, *p* = 0.008, followed by Bonferroni’s *post hoc* test, ***p < 0.001). All data are presented as mean ± SEM. See also Extended Data Figure 1-1.

10.1523/ENEURO.0370-20.2020.f1-1 Extended Data Figure 1-1 *Oprk1* signal intensity in CeA subdivisions and post-threat conditioning EPM in *Oprk1* KO mice. ***A***, Representative image of *Oprk1* mRNA in the CeA. Scale bar: 100 μm. ***B***, Average pixel intensity of *Oprk1* mRNA fluorescent *in situ* signal in subdivisions of the CeA along the rostral to caudal axis (two-way ANOVA, *F*_(6,27)_ = 3.87, *p* < 0.01, followed by Bonferroni’s multiple comparisons, *p < 0.05, ***p < 0.001). ***C***, Binned *Oprk1* transcript per cell in controls and *Oprk1* KOs (two-way ANOVA, *F*_(9,63)_ = 2.541, *p = 0.0148). ***D***, ***E***, Average freezing response during 10 trials of day 2 in control and *Oprk1* KO mice during CS+ (***D***, Student’s *t* test, *p* < 0.05) and CS– (***E***). ***F***, Time spent in open and closed arms of the EPM in control and *Oprk1* KO mice following threat conditioning (control, *N* = 13; *Oprk1* KO, *N* = 7). ***G***, [1-(open/closed arm time)] in control and *Oprk1* KO mice following threat conditioning. Data are presented as mean ± SEM. Download Figure 1-1, TIF file.

### Fear conditioning

Fear conditioning sessions were performed during the light cycle in a standard operant chamber (Med Associates Inc.) equipped with a house light, tone generator, and shock grid. Our discriminative low threat intensity fear conditioning protocol is 3 d long and includes a baseline probe then shock training trial on day 1, an intermediate probe and second shock training trial on day 2, and a final probe on day 3.

During each training session, animals are exposed to 10 pairings of a CS+ auditory tone with a 0.3-mA US footshock, and 10 CS– tone presentations which are never paired with shock. Auditory CS cues included a 10-kHz pulsatile tone and a 20-kHz continuous tone, each 10 s in duration. The CS+ tone co-terminated with a 0.5-s 0.3-mA footshock which began 9.5 s after the tone presentation began. Assignment of which tone would be the shock-paired CS+ or non-shock paired CS– was counterbalanced across groups. CS+ and CS– tone presentations alternated with a 60-s intertrial interval. The training context was all metal, with the shock grid floor, and the box was cleaned with 70% ethanol between trials.

Baseline and probe trials were tested in a separate context, in an all-white Med-Associates box insert with a smooth floor which was cleaned with a 1% acetic acid between animals. The animals were exposed to alternating presentations of 3 CS+ and 3 CS– auditory tones (10-s tones as described above with a 60-s intertrial interval, but with no shock presentations). These trials were used to establish basal freezing to the tones on day 1 before shock training and to measure expression of learned fear of the tone(s).

All sessions were video recorded, and nose, center, and tail points were tracked on each mouse using Ethovision (Noldus) tracking software. Velocities were calculated using an in-house MATLAB (Mathworks) script, which calculates the animal’s velocity given the movement of all three points. Freezing was defined as velocity <1 cm/s that lasted ≥0.5 s. “% freezing” was calculated as the % of time during the 10-s CS tone that the animal was frozen. Probe trial % freezing was presented as an average response to the 3 CS+ tones and the 3 CS– tones for each experimental group during the day 1 baseline and the day 3 final probe. Training/acquisition freezing was presented as the 20 total CS+ or CS– trials, binned into an average of two trials (ex: average of trials 1 and 2, 3, and 4, etc.). To measure shock reactivity, the raw center point velocity from Ethovision was graphed from 1 s before to 3 s after shock, for the first 10 shock trials.

### Histology and immunostaining

Mice were anesthetized with 50 mg/kg of Beuthanasia-D and transcardially perfused with PBS, followed by 4% paraformaldehyde until the tissue was firm. Whole-brain tissue was dissected out and fixed overnight in 4% paraformaldehyde, and then cryoprotected by soaking in a 30% sucrose in PBS for 48 h. The brains were frozen in OCT in a −20°C cryostat and then cryosection to produce 30-μm-thick sections. Brain sections of interest were collected in PBS with 0.1% NaAz before immunostaining.

For whole-brain retrograde labeling experiments, every section was collected, and a section was stained for every 90 μm (approximately corresponding to one page in the Paxinos atlas), or every 180 μm for projection analysis. For CeA-specific immunostaining, sections were selected based on the Paxinos and Franklin reference atlas, selecting sections which cover the length of the CeA (y = −1.2 to −1.18.).

Selected sections to stain were washed in tris-buffered saline (TBS) for 10 min then blocked in TBS + 0.3% Triton X-100 (TBST) +3% normal donkey serum (NDS) for 30 min. Then sections were incubated overnight in primary antibody diluted in TBST+NDS. Anti-GFP (polyclonal, 1:2000; Invitrogen A11122; polyclonal, 1:1000, Abcam ab13970) was used to stain for GFP and eYFP-tagged viruses and anti-dsRed (polyclonal, 1:2000; Clontech, 632496) was used for mCherry-tagged viruses.

CeA Pdyn^+^ synaptophysin distal projections were quantified by collecting the whole brain then staining sections (for GFP, as described above) every 180 μm. Because of GFP expression in the cell bodies in the CeA, the density of projections in the CeA could not be quantified. Six extra-amygdalar projections were identified and quantified by drawing regions of interest (ROIs) around the target region. We measured the density of GFP-tagged projections by measuring the sum of the values of the pixels in the ROI (RawIntDen) and dividing that by the area of the ROI.

For the Pdyn^+^ retrograde projection labeling experiment, cells were counted throughout the whole brain. The location of labeled cells was determined using the Paxinos and Franklin mouse brain atlas. Normalized cell count per region was calculated as [region count/(mouse total labeled cells/average total cells)].

### RNAscope *in situ* hybridization

The RNAscope assay (Wang et al., 2012) performed on Bl6 wild-type, *Oprk* lox/lox, and *Pdyn* lox/lox adult mice. Viral vectors were injected (as described above) two to three weeks before brains were quickly excised, flash frozen in 2-methylbutane, and stored at −80°C; 20-μm brain slices were prepared spanning the CeA from −1.2 to 1.8 mm posterior of bregma (according to the Paxinos atlas). Sections were prepared for hybridization per manufacturer’s (Advanced Cell Diagnostics, Inc) instructions using probes for *Cre* (Mm-*Cre*-C2, ACD bio #312281-C2), Pdyn (Mm-*Pdyn*-C3, ACD bio #318771), and Oprk (Mm-*Oprk*-C3, ACD bio #316111-C2). Slides were coverslipped and imaged using Nikon Upright Widefield fluorescent microscope at 10× magnification. Analysis performed using ImageJ.

Paxinos and Franklin’s mouse brain atlas was used to determine CeA and subregion locations. Imaging exposure was chosen based on a reference area which did not receive Cre-GFP (the basolateral amygdala for *Oprk*, and the caudate putamen for *Pdyn*). Expression in the CeA and KO was measured as number of cells in the CeA-expressing the gene of interest. Gene expression was quantified from y = −1.23 to −1.79 (atlas pages 41–46), and KO was quantified over five sections from y = −1.31 to −1.79 for Oprk and six sections from y = −1.23 to −1.79 for Pdyn, based on location of gene expression. A cell was determined to express the gene of interest if there were more than three transcript puncta associated with the same nucleus (visualized using DAPI Fluoromount-G). Cell counting and KO quantification was performed for each unilateral injection. After noting differences in transcript expression level per cell between CeA subregions, we compared the density of transcript being expressed per subregion using ImageJ. Pixel density was calculated by measuring the sum of the values of the pixels in the ROI (RawIntDen) divided by the area of the ROI.

Additional analysis of KO was performed on 10× images using CellProfiler software, to assess gene expression using integrated pixel intensity per cell. Nuclei (primary objects) were identified using DAPI (threshold strategy: global, thresholding method: two-class Otsu, method for distinguishing clumped objects: intensity, dividing lines between clumped objects method = propagate) and cells (secondary objects) were identified as the nucleus expanded by a distance of three pixels.

## Results

### KOR signaling in the CeA regulates anxiety-like behavior and discriminative threat conditioning

Previous studies of mRNA expression indicate that a fraction of cells in the CeM and CeL express *Oprk1* (Zhu and Pan, 2004; Chieng et al., 2006; Bloodgood et al., 2020). KOR activation has also been shown to reduce synaptic transmission throughout the CeA and hyperpolarize a portion of CeM cells (Zhu and Pan, 2004; Chieng et al., 2006; Kang-Park et al., 2013). To better resolve the distribution of *Oprk1* expression in the CeA we used RNAscope fluorescent *in situ* hybridization to probe for *Oprk1* mRNA. *Oprk1* was found to be expressed in all three subregions, with the most *Oprk1*^+^ cells in the CeM (Fig. 1*A–C*). Although the CeM possessed the largest number of *Oprk1*^+^ cells, the highest levels of expression were observed in the CeL (Extended Data Fig. 1-1*A*,*B*).

To determine whether KOR signaling in the CeA regulates anxiety-like behavior and conditioned threat discrimination, we inactivated *Oprk1* by injecting *Oprk1^lox/lox^* mice with AAV1-Cre-EGFP bilaterally into the CeA (Fig. 1*D*). Because of the high levels of *Oprk1* expression in the adjacent BLA and the known role of BLA KOR in regulating fear and anxiety (Knoll et al., 2011), we used a dilute virus and excluded all mice with viral spread into adjacent structures. This resulted in a significant reduction in *Oprk1* mRNA levels across the entire rostral to caudal CeA (Fig. 1*E*,*F*), although the effect was modest given the stringent criterion for inclusion. Partial KO of *Oprk1* in the CeA (*Oprk1 KO*) resulted in a significant increase in anxiety-like behavior as measured by time spent in the closed arms of an EPM and a shift in the fraction of time spent in the closed versus open arms [graphed as 1-(open arm time/closed arm time) so that an increase in anxiety-like behavior is reflected by an increase in this fraction; Fig. 1*G–I*].

Twenty-four hours following testing in the EPM, mice were assessed in a discriminatory threat conditioning paradigm (Fig. 1*J*). Before conditioning, baseline freezing was measured in response to three presentations of two distinct tones that would be used for pairing with the US during conditioning (CS+) or that would be presented during conditioning but unpaired with the US(CS–). During conditioning, mice were trained for to two consecutive days in a different context than the probe test with 10 pairings of the CS+ tone that co-terminated with a 0.3-mA footshock (0.5 s), and 10 CS– tone presentations. Twenty-four hours following the last training session, mice were probed for retention by measuring freezing in response to three presentations of the CS+ and CS– tones. During acquisition training, both CeA *Oprk1* KO and control mice responded equivalently to the US (Fig. 1*K*). Neither group displayed discrimination between the CS+ and CS– during conditioning (Fig. 1*L*,*M*); however, *Oprk1* KO mice had higher freezing to the CS+ on average during the second day of conditioning compared with control mice (Extended Data Fig. 1-1*C*,*D*). During the probe for retrieval 24 h postconditioning, control mice showed robust discrimination between the CS+ and CS– (Fig. 1*N*) that was not observed in the CeA *Oprk1* KO mice (Fig. 1*O*).

Following fear conditioning, we assessed behavior in the EPM again in a subset of mice to investigate whether there were differential effects of threat conditioning stress on anxiety-like behavior. In this context, we still observed a slightly higher time spent in the closed arms; however, this effect was no longer significant because of increased anxiety-like behavior in control mice after fear conditioning (Extended Data Fig. 1-1*E*,*F*).

### *Pdyn* expression in the CeA does not regulate anxiety-like behavior or threat discrimination

**Figure 2.**
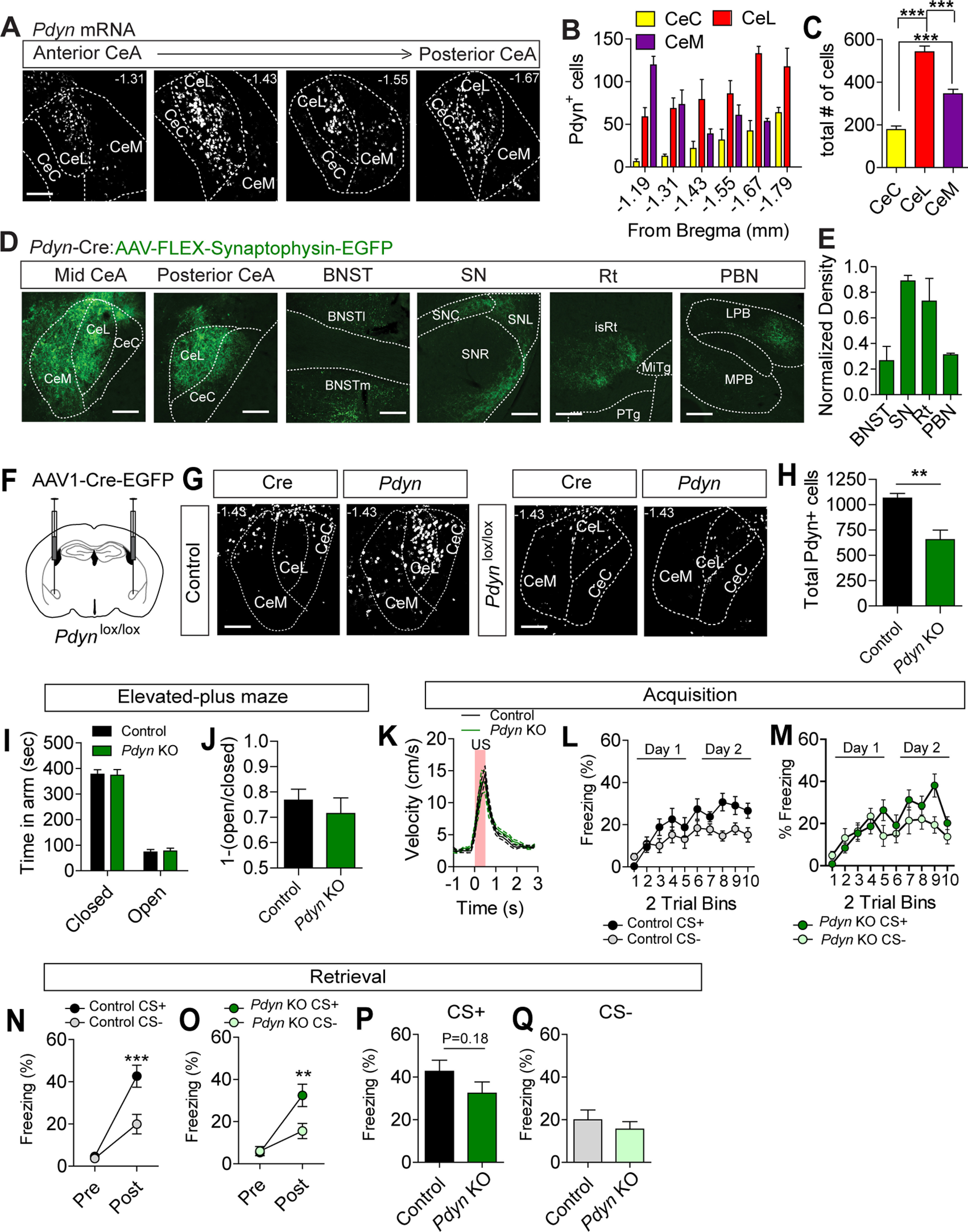
CeA *Pdyn* expression and regulation of anxiety-like behavior and threat. ***A***, Representative images of *Pdyn* mRNA expression along the rostral-caudal extent of the CeA. Scale bar: 100 μm. ***B***, Distribution of cells along the rostral-caudal extent of the CeA-expressing *Pdyn* (*N* = 5). ***C***, Distribution of cells expressing *Pdyn* within the subdivisions of the CeA (one-way ANOVA, *F*_(2,12)_ = 67.34, *p* < 0.0001, followed by Tukey’s *post hoc* comparison test, ***p < 0.001). ***D***, Expression of Synaptophysin-EGFP in the CeA and projection targets of *Pdyn^Cre/+^* mice. Scale bar: 100 μm. ***E***, Quantification of normalized projection densities for Synaptophysin-EGFP levels in *Pdyn^Cre/+^* mice injected with AAV1-FLEX-Synaptophysin-EGFP into the CeA (*N* = 3 mice). BNST, bed nucleus of the stria terminalis; SN, substantia nigra; Rt, pontine reticular formation; PBN, parabrachial nucleus. ***F***, Schematic of injection for AAV1-Cre-GFP into the CeA of *Pdyn^lox/lox^* mice. ***G***, *Pdyn* mRNA in the CeA of control (*Pdyn^+/+^*) and *Pdyn^lox/lox^* mice. Scale bar: 100 μm. ***H***, Quantification of *Pdyn* mRNA-positive cells in the CeA following Cre expression in control (*N* = 5) and *Pdyn^lox/lox^* (*N* = 3) mice (Student’s *t* test, **p < 0.01). ***I***, Time spent in open and closed arms of the EPM in control (*N* = 9) and *Pdyn* KO (*N* = 9) mice. ***J***, [1-(open/closed arm time)] in control and *Pdyn* KO mice. ***K***, Average US responses in control (*N* = 14) and *Pdyn* KO mice (*N* = 14) during training. ***L***, ***M***, Average CS+ and CS– freezing in control (***L***) and *Pdyn* KO mice (***M***) during acquisition. ***N***, ***O***, Average freezing to CS+ and CS– during probe trials for retrieval in control (***N***, *N* = 17) and Pdyn KO mice (***O***, *N* = 14). Both control and *Pdyn* KO mice showed significant cue discrimination during probe trials (control: two-way ANOVA, *F*_(1,32)_ = 6.51, *p* = 0.004, followed by Bonferroni’s *post hoc* test, ***p < 0.001; *Pdyn* KO: two-way ANOVA, *F*_(1,26)_ = 7.05, *p* = 0.008, followed by Bonferroni’s *post hoc* test, ***p < 0.001). ***P***, ***Q***, No significant effects of *Pdyn* KO were observed on freezing to the CS+ (***P***, Student’s *t* test, *p* = 0.18) or CS– (***Q***, Student’s *t* test, *p* = 0.47). All data are presented as mean ± SEM. See also Extended Data Figure 2-1.

Given the high degree of interconnectivity of neurons within the CeA (Ciocchi et al., 2010), we hypothesized that inactivation of local dynorphin production in the CeA from the *Pdyn* gene would phenocopy inactivation of *Oprk1* in the CeA. To confirm *Pdyn* expression in the CeA and delineate its anatomic distribution, we visualized *Pdyn* mRNA using *in situ* hybridization. *Pdyn* mRNA was localized throughout the rostral-to-caudal extent of the CeA with the highest numbers of *Pdyn*^+^ cells in the CeL (Fig. 2*A–C*), as was previously reported (Kim et al., 2017). To determine the projections of CeA *Pdyn*^+^ cells, we performed viral projection mapping using *Pdyn^Cre/+^* mice (Knoll et al., 2011). First, we confirmed the efficacy of viral labeling in the CeA by injecting *Pdyn^Cre/+^*mice with AAV1-FLEX-mCherry. Consistent with our *in situ* analysis, we observed robust viral labeling in the CeA of *Pdyn^Cre/+^* mice (Extended Data Fig. 2-1*A*,*B*). Next, we injected AAV1-FLEX-Synaptophysin-EGFP into the CeA of *Pdyn*^Cre/+^ mice to visualize neuronal projections. We observed dense synaptophysin-GFP labeling within the CeL and CeM, as well as projections outside the CeA to the BNST, substantia nigra (SN), the midbrain reticular nucleus (Rt), and the LPN (Fig. 2*D*,*E*).

10.1523/ENEURO.0370-20.2020.f2-1 Extended Data Figure 2-1 Viral labeling of *Pdyn-*expressing cells in the CeA and post-threat conditioning EPM in *Pdyn* KO mice. ***A***, Binned *Pdyn* transcript per cell in controls and *Pdyn* KOs (two-way ANOVA, *F*_(19,114)_ = 9.011, ****p < 0.0001, followed by Bonferroni’s *post hoc* test, ****p < 0.0001, **p < 0.01). ***B***, Representative images of mCherry expression in the CeA of *Pdyn^Cre/+^* mice. Scale bar: 100 μm. ***C***, Quantification of mCherry-expressing cells in the CeA of *Pdyn^Cre/+^* mice. ***D***, ***E***, Average freezing response during 10 trials of day 2 in control and *Pdyn* KO mice during CS+ (***D***) and CS– (***E***). ***F***, Time spent in open and closed arms of the EPM in control and *Pdyn* KO mice following threat conditioning (control, *N* = 9; *Pdyn* KO, *N* = 9). ***G***, [1-(open/closed arm time)] in control and *Pdyn* KO mice following threat conditioning. Data are presented as mean ± SEM. Download Figure 2-1, TIF file.

To determine whether inactivation of *Pdyn* from neurons within the CeA is sufficient to phenocopy the inactivation of *Oprk1* in this region, injected AAV1-Cre-EGFP bilaterally into the CeA of *Pdyn*^lox/lox^ mice (Fig. 2*F*). Using a similar strict exclusion criterion as described for *Oprk1* inactivation, we observed a significant reduction in *Pdyn* mRNA levels in the CeA (Fig. 2*G*,*H*) which was comparable to CeA *Pdyn* KO reported elsewhere (Bloodgood et al., 2020). In contrast to the observations made following a reduction in *Oprk1* levels, a reduction in *Pdyn* did not alter behavior in the EPM (Fig. 2*I*,*J*). Likewise, we did not observe any significant effect of *Pdyn* reduction in the CeA on discriminatory threat conditioning during either acquisition (Fig. 2*K–M*; Extended Data Fig. 2-1*C*,*D*) or retrieval (Fig. 2*M–O*). In rats, knock-down of *Pdyn* expression with shRNA in *Crh*-expressing neurons of the CeA reduces freezing to a CS+ (Pomrenze et al., 2019). Although we observed a reduction in CS+-evoked freezing, this effect was not significant (Fig. 2*P*,*Q*). We also did not observe significant effects in the EPM in post-threat conditioned mice (Extended Data Fig. 2-1*E*,*F*).

### Inactivation of *Pdyn* from local and distal sources increases anxiety-like behavior and reduces threat discrimination

**Figure 3.**
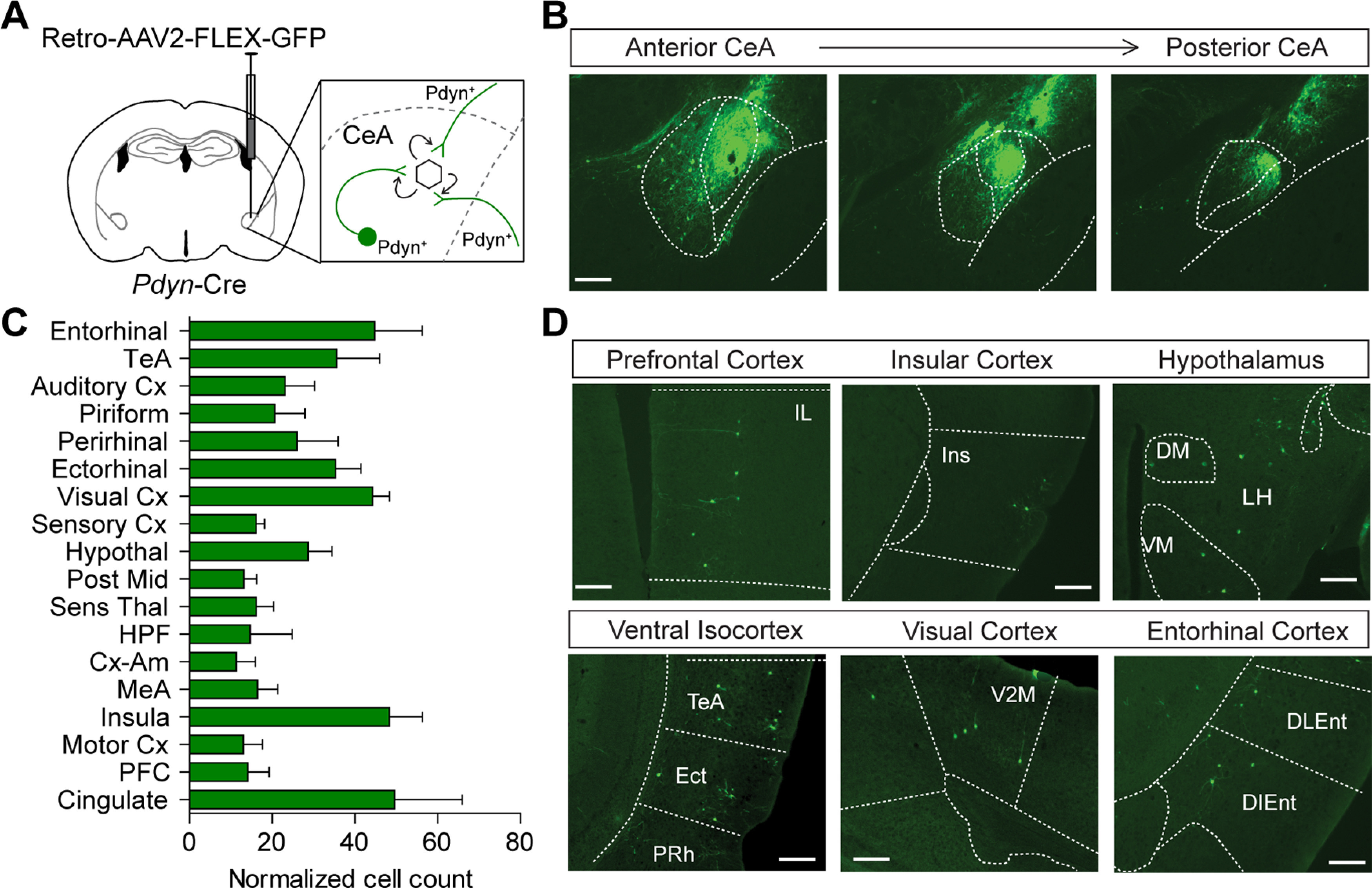
*Pdyn* inputs to the CeA. ***A***, Schematic of Retro-AAV2-FLEX-EGFP injection into the CeA of *Pdyn^Cre/+^* mice. ***B***, Representative images of EGFP expression in the along the anterior-posterior axis of the CeA. Scale bar: 100 μm. ***C***, Distribution of relative cell numbers outside the CeA-expressing EGFP (*N* = 3 mice). ***D***, Representative images of *Pdyn^Cre^-*positive neurons expressing EGFP, largely in the cortex and hypothalamus. Scale bar: 100 μm. TeA, temporal association cortex; Cx, cortex; hypothal, hypothalamus; Mid, midbrain; sens thal, sensory thalamus; HPF, hippocampal formation; Cx-Am, cortex-amygdala transition zone; MeA, medial amygdala; PFC, prefrontal cortex. Data are presented as the mean ± SEM. See also Extended Data Figure 3-1.

One potential explanation for the lack of an observed effect of *Pdyn* reduction on discriminatory threat conditioning and anxiety-like behavior is that dynorphin is also released from inputs to the CeA that arrive from outside the structure. To investigate whether there are distal sources of dynorphin inputs to the CeA, we injected a retrograde transducing virus with a Cre-conditional expression cassette for EGFP (Retro-AAV2-FLEX-EGFP) unilaterally into the CeA of *Pdyn^Cre/+^* mice (Fig. 3*A*). We observed EGFP expression throughout the anterior-posterior axis of the CeA (Fig. 3*B*). The majority of the injections were restricted to the CeA, although we did observe a small amount of virus in the striatum along the injection tract (Extended Data Fig. 3-1*A*). Retrogradely labeled cells were observed in multiple brain regions, but predominantly in cortical and hypothalamic areas. The largest number of labeled cells were in the isocortex, cingulate, insular cortex, entorhinal cortex, and hypothalamus (Fig. 3*C*,*D*; Extended Data Fig. 3-1*B*).

10.1523/ENEURO.0370-20.2020.f3-1 Extended Data Figure 3-1 Tissue distribution of Retro-AAV2-EGFP for mapping *Pdyn*-expressing inputs to the CeA. ***A***, Cartoon images of mouse brain section along the rostral to caudal axis containing the CeA. Individual mice are pseudocolored to display viral spread at the injection site. ***B***, Distribution of the percentage of EGFP-expressing neurons in individual mice in brain regions with *Pdyn*-expressing projections to the CeA. Cx-Amyg, cortex-amygdala transition zone; Post Midb, posterior midbrain; Cx-Motor, motor cortex; Sens-Thal, sensory thalamus; HPF, hippocampal formation; hypothal, hypothalamus; mPFC+Cg, medial prefrontal cortex and cingulated cortex. Download Figure 3-1, TIF file.

**Figure 4.**
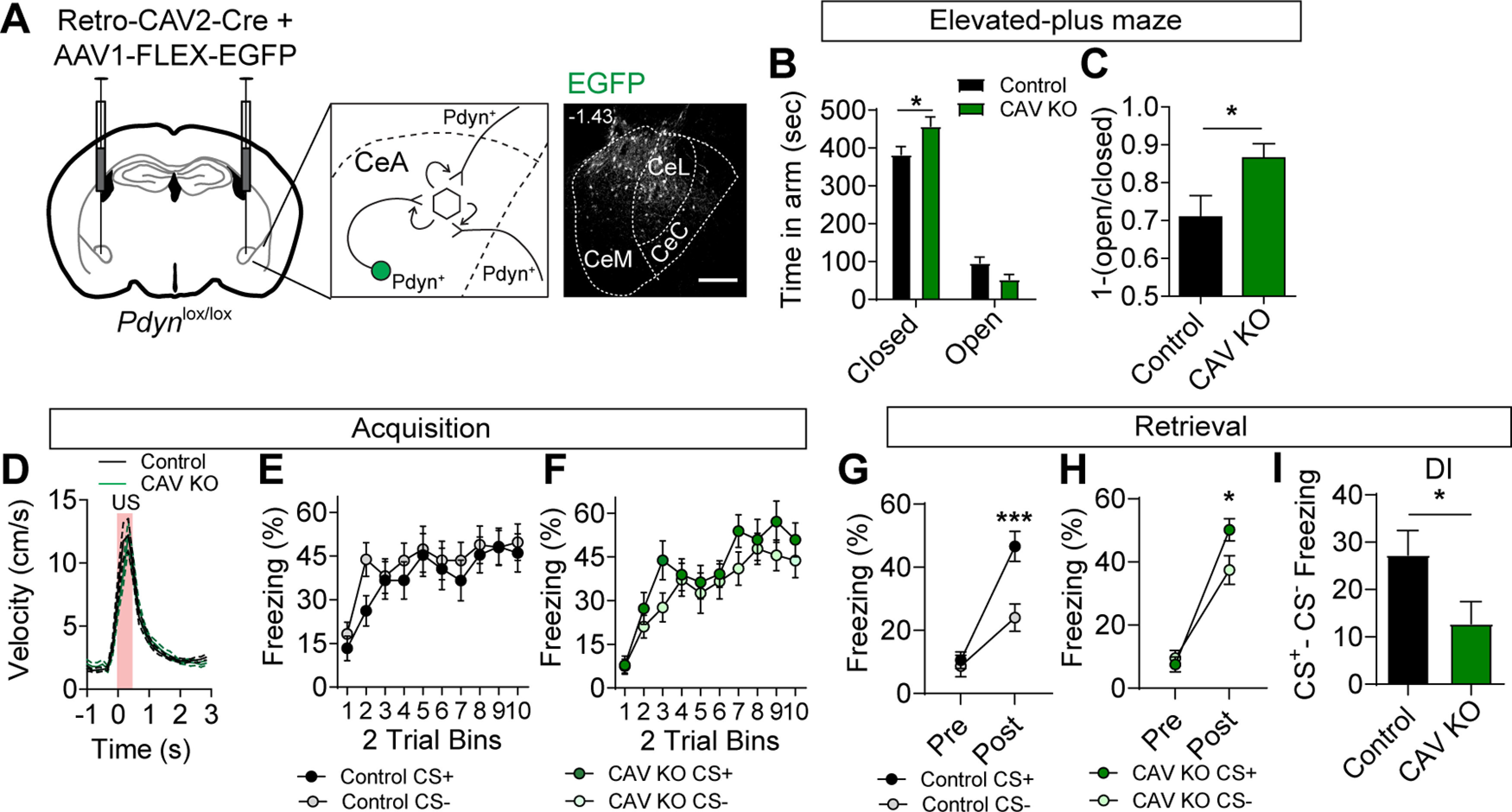
Inactivation of *Pdyn* in inputs to the CeA. ***A***, Schematic of injection for Retro-CAV2-Cre and AAV1-FLEX-EGFP into the CeA of *Pdyn^lox/lox^* mice (left) and corresponding representative image of EGFP expression in the CeA. Scale bar: 100 μm. ***B***, Time spent in open and closed arms of the EPM in control (*N* = 15) and CAV KO mice (*N* = 11; two-way ANOVA, *F*_(3,72)_ = 3.95, *p* = 0.012, followed by Bonferroni’s *post hoc* test, *p < 0.05). ***C***, [1-(open/closed arm time)] in control and CAV KO mice (Student’s *t* test, *p < 0.05). ***D***, Average US responses in control (*N* = 17) and CAV KO mice (*N* = 14) during training. ***E***, ***F***, Average CS+ and CS– freezing in control (***E***) and CAV KO mice (***F***) during acquisition (control, *N* = 17; CAV KO, *N* = 14). ***G***, ***H***, Average freezing to CS+ and CS– during probe trials for retrieval in control (***G***) and CAV KO mice (***H***). Both control and Pdyn KO mice showed significant cue discrimination during probe trials (control: two-way ANOVA, *F*_(1,32)_ = 8.00, *p* = 0.0002, followed by Bonferroni’s *post hoc* test, ***p < 0.001; CAV KO: two-way ANOVA, *F*_(1,26)_ = 2.87, *p* = 0.046, followed by Bonferroni’s *post hoc* test, *p < 0.05). ***I***, Discrimination index [(CS+) – (CS–)] is significantly reduced in CAV KO mice relative to controls (Student’s *t* test, *p < 0.05). All data are presented as mean ± SEM. See also Extended Data Figure 4-1.

To determine whether inactivation of *Pdyn* expression from a diversity of inputs to the CeA is sufficient to phenocopy the inactivation of *Oprk1* on anxiety-like behavior and conditioned threat discrimination, we injected *Pdyn^lox/lox^* and control (*Pdyn^+/+^*) mice with the retrograde transducing virus CAV2 containing an expression cassette for Cre recombinase (CAV2-Cre). To confirm the locations of the injection sites, we co-injected mice with an AAV containing a Cre-dependent expression cassette for EGFP (AAV1-FLEX-EGFP; Fig. 4*A*). Unlike *Pdyn^lox/lox^* mice injected with AAV1-Cre-EGFP to KO local *Pdyn* in the CeA*, Pdyn^lox/lox^* mice injected with CAV2-Cre and AAV1-FLEX-EGFP (CAV KO) largely phenocopied *Oprk1* inactivation. CAV KO mice had a significant increase in anxiety-like behavior, as measured by an increased time in the closed arms and a trending decrease in time spent in the open arms of the EPM, relative to controls (Fig. 4*B*,*C*).

10.1523/ENEURO.0370-20.2020.f4-1 Extended Data Figure 4-1 Post-threat conditioning EPM in CAV KO mice. ***A***, ***B***, Average freezing response during 10 trials of day 2 in control and CAV KO KO mice during CS+ (***A***) and CS– (***D***). ***C***, Time spent in open and closed arms of the EPM in control and CAV KO mice following threat conditioning (control, *N* = 15; CAV KO, *N* = 11). ***D***, [1-(open/closed arm time)] in control and CAV KO mice following threat conditioning. Data are presented as mean ± SEM. Download Figure 4-1, TIF file.

During acquisition training in the discriminative threat conditioning paradigm, CAV KO and control mice showed similar US responses and freezing to CS+ and CS– tones during conditioning (Fig. 4*D–F*). Unlike *Oprk1* reduction in the CeA, we did not observe significant effects of CAV KO on freezing during the acquisition (Extended Data Fig. 4-1*A*,*B*). During retrieval, both control and CAV KO mice had significant threat discrimination in the 24 h probe trial (Fig. 4*G*,*H*); however, the discrimination index (CS+ minus CS–) was significantly reduced in CAV KO mice relative to controls (Fig. 4*I*) consistent with an increase in threat generalization. Like other groups, threat conditioning enhanced anxiety-like behavior similarly in control and CAV KO mice (Extended Data Fig. 4-1*C*,*D*).

## Discussion

It has been well established that dynorphin and KOR play a critical role in response to stress and that the net effect of global KOR activation is dysphoria and anxiety (Pfeiffer et al., 1986; Nabeshima et al., 1992; McLaughlin et al., 2003; Land et al., 2008). This has led to the assumption that dynorphin acts as prostress molecule in all circuits. Our results suggest a more complex function of KOR-dynorphin signaling in which KOR signaling in CeA neurons promotes threat discrimination and is anxiolytic. Consistent with this notion, acute KOR activation results in a variety of adaptive responses to threat, including analgesia which likely increases an organism’s ability to cope or escape from noxious stimuli (Knoll and Carlezon, 2010; Chavkin, 2013). Additionally, dynorphin in the NAc suppresses attentional responding to non-predictive cues, further supporting a role of KOR-dynorphin signaling in discriminatory behavior (Iordanova et al., 2006). Dysphoria and aversion, which is associated with a threat, motivates the organism to engage in behaviors to avoid the hazard in the future (Knoll and Carlezon, 2010).

Dynorphin is a signaling peptide and its effects are dependent on the localization of it inhibitory receptor, KOR. It has been previously demonstrated that dynorphin-KOR activation in adjacent subregions can result in opposing behavioral outcomes. It was demonstrated that stimulation of dynorphin populations in the ventral NAc shell was aversive, while stimulation of dynorphin cells in the adjacent dorsal NAc shell mediated place-preference and was positively reinforcing (Al-Hasani et al., 2015). Additionally, global *Pdyn* KO experiments resulted in enhancement of amygdala-mediated cued fear, but not hippocampus-mediated contextual fear (Bilkei-Gorzo et al., 2012).

In the CeA, previous work has shown that CeA KOR and CeA CRF receptor signaling have opposing effects on physiology in the CeA (Kang-Park et al., 2015). Given that CRF in the CeA is known to enhance threat responses (Fadok et al., 2017; Sanford et al., 2017; Jo et al., 2020), it is not surprising then that KOR suppresses threat responsiveness in this context. We observed *Oprk1* expression to be widespread in the CeA with the highest number of *Oprk1^+^* cells in the CeM with the highest density of *Oprk1* mRNA levels in the CeL. It has also been shown that a proportion of CeL *Oprk1-*expressing neurons have overlapping expression with *Pdyn* (Bloodgood et al., 2020) and dynorphin co-localizes with SOM-expressing “fear on” neurons (Haubensak et al., 2010; Li et al., 2013; Kim et al., 2017; Ahrens et al., 2018). Thus, KOR-mediated inhibition of the CeL fear on neurons in addition to CeM output neurons would be predicted to lead to a decrease in overall CeA threat-related output signals.

The effects of reducing KOR in the CeA on basal anxiety-like behavior was not previously identified (Bloodgood et al., 2020). One possible explanation for this discrepancy is that the previous study performed their alcohol-naive, basal anxiety assays in mice that had been singly housed for one to three weeks, whereas our mice are group housed. Social isolation stress has been reported to modify neural circuits regulating anxiety and to increase anxiety-like behavior (Da Silva et al., 1996; Matthews et al., 2016). Our assessment of anxiety-like behavior in mice following threat conditioning demonstrates that stress can mask basal differences observed in stress-naive mice. These authors also looked at sex-specific effects on alcohol consumption. In our data, there were no obvious differences in behavioral trends between males and females. For every experiment performed, male and female data were separated and assessed for trending differences, of which none were found, so male and female data were combined.

The role of dynorphin in the CeA for the regulation of alcohol consumption has been well described (Gilpin and Roberto, 2012; Anderson et al., 2019; Bloodgood et al., 2020). Several studies have also investigated the role of KOR-dynorphin in the CeA for the regulation of fear and anxiety. Using a fear-potentiated startle paradigm where rats receive KOR antagonist infusions between postfear-conditioning startle sessions, pharmacological inhibition of KOR in the CeA reduced conditioned startle (Knoll et al., 2011). It is not entirely surprising that their pharmacological manipulation of conditioned startle and our genetic manipulation of cued conditioned freezing implicated divergent roles for CeA KOR. Behavior task differences aside, we inactivated *Oprk1* only in CeA neurons, whereas antagonist infusion blocks KOR signaling at the level of the cells in the CeA as well as on terminals of neurons projecting into the CeA.

In addition to KOR, dynorphin has also been studied in the CeA in the context of fear-related and anxiety-related behavior. Dynorphin is increased in SOM-expressing neurons in the CeA in a mouse model of anxiety that is proposed to increase KOR-dynorphin in the BNST to promote increased anxiety-like behaviors (Ahrens et al., 2018). We did not observe a change in anxiety-like behavior following conditioning in CeA *Pdyn* KO mice, consistent with previous assessment of CeA-*Pdyn* KO in the EPM (Bloodgood et al., 2020). It has also been shown that dynorphin KO in CeA CRF neurons in rats reduces freezing during probe trials (Pomrenze et al., 2019). We observed a modest reduction in freezing to the CS+ that did not reach significance although the reductions in *Pdyn* levels are comparable. This difference could be because of the manipulation of slightly different dynorphin populations, non-selective here versus *Crh-*selective in the rat. These differences could also be a reflection of the magnitude of the US, we use a moderate US that promotes discrimination in mice whereas the US used in the previous study in higher and results in a higher level of overall freezing. Thus, the effects of dynorphin produced in the CeA may only become evident at higher threat levels. Regardless of these minor differences, our findings reducing local *Pdyn* expression in the CeA does not phenocopy a reduction in *Oprk1* levels in the CeA. This implies that a distal source of dynorphin, or a combination of local and distal dynorphin are required to signal through KOR within the CeA to suppress basal anxiety levels and promote conditioned threat discrimination. Our retrograde tracing suggests that the external sources of dynorphin could be cortical or hypothalamic and future investigations using combinatorial strategies should illuminate which of these inputs are critical, although it should be noted that the broad distribution of *Pdyn*^+^ cells projecting to the CeA will make this a challenging task.

## Synthesis

Reviewing Editor: Carmen Sandi, Swiss Federal Institute of Technology Decisions are customarily a result of the Reviewing Editor and the peer reviewers coming together and discussing their recommendations until a consensus is reached. When revisions are invited, a fact-based synthesis statement explaining their decision and outlining what is needed to prepare a revision will be listed below. The following reviewer(s) agreed to reveal their identity: Julia Lemos.

The two reviewers and the review editor find this manuscript interesting and relevant. It is well designed, the analyses and results are sound, and the manuscript is well written and easy to follow. It demonstrates that the dynorphin-kappa opioid system is not a monolith, but rather has a novel role in the central amygdala to discriminate threatening cues and promote exploratory behavior. Specifically, it provides new mechanisms and KOR projections in the CeA in relationship to anxiety-like behavior and safety learning.

The study focuses on the role of dynorphin-kappa opioid receptors (KOR) in the central nucleus of the amygdala (CeA) in regulating anxiety-like behavior and threat discrimination. To this end, the authors manipulated both kappa opioid and dynorphin levels in the CeA using a combination of viral strategies and transgenics. The results indicate that dynorphin inputs - likely from the cortex and hypothalamus - to the CeA also regulate anxiety-like behavior and have a role in a threat discrimination index. As a result, the study suggests an interesting dynorphin circuit from the cortex and hypothalamus to CeA which may project to BNST, SN, RT and PBN.

Despite the many merits of the study, there are several issues that need to be addressed before publication:

1. The ms. refers to CeC as central part of the central amygdala (CeA). However, the second edition of “The Mouse Brain in Stereotaxic Coordinates” (Paxinos & Franklin) and the Allen Brain Atlas name CeC as capsular part of the CeA. This needs to be corrected.

2. Anxiety index: The parameter and terminology used are unconventional. We recommend to use the customary ones in the literature (i.e., % open arm time). Note, in addition, that the term “anxiety” to refer to animal behavior is considered by a large part of the neuroscience community as too anthropomorphic.

3. State what the control viruses were for each experiment.

4. RNAscope experiments: We recommend to sample CeA cells from control and KOR or pDyn knock-down conditions (Figure 1f, 2h) and measure the number of puncta per cell. The knock-down should reduce the number of transcripts and it may be a more robust knock-down when the number of puncta is assessed.

5. State the age range of the mice.

6. Under “Virus production and surgery” in the Methods - “produced by XXXX and was a gift of XXXX” should be corrected.

7. The ms. discusses the paper from Bloodgood et al. 2020 in relation to the reported anxiety-like behavioral results. While we find the given interpretation agreeable (it is known that single housed and group housed animals can behave quite differently), it would be important to include in this discussion that in the Bloodgood et al. 2020 article, the behavior under study is different (i.e., anxiety-like behavior elicited by ethanol consumption which can be different from the present study in naïve animals). A plausible interpretation offered by one of the reviewers of this manuscript is that “both datasets is that the KOR-dynorphin signaling in the CeA is important in healthy animals but not in animals that have been exposed to ethanol consumption with subsequent stress”.

8. There are sex-specific effects in the CeA of KOR and preprodynorphin (PDYN) shown in Bloodgood et al. 2020. The use of both male and female mice is a strong point of the present manuscript. However, the current study does not seem to have an N per group large enough to perform analyses by sex, which would greatly increase the relevance of this work. It would be important to address this issue, if not with new experiments, with careful consideration in the Discussion section.

9. Interestingly, the ms. reports that reduction of both KOR in the CeA and dynorphin from distil inputs into the CeA lead to an increase in anxiety-like behavior assessed via elevated plus maze and threat discrimination assessed via the conditioned fear paradigm. While this is surprising when considering it in the context of the entire field, it is not without precedent. A recent paper by Al-Hasani et al., 2015, Neuron demonstrated that dynorphin release can produce a place preference or aversion depending on what aspect of the NAc shell is being stimulated. This article should be cited, as it is very relevant in the context of the current study and it provides support for appetitive behaviors that are encoded by dynorphin.

10. The discussion of the paper by Knoll et al. 2011 should also include that the fear task used is different from the one the authors of the present manuscript have tested.

11. While the discussion includes previous papers that have studied fear/threat in KOR-Dynorphin neurons in the CeA, it is missing some important KOR-Dynorphin studies focusing on auditory fear conditioning which heavily rely on the CeA. E.g. PMID: 22764240.

12. Figure 2B and Extended Figure 2B. The authors may want to consider modifying X axis coordinates to make them equivalent (currently different).
