# Supplementary figures and images for: κ Opioid Receptor-Dynorphin Signaling in the Central Amygdala Regulates Conditioned Threat Discrimination and Anxiety
Source: eNeuro. 2021 Jan 12;8(1):ENEURO.0370-20.2020. doi: 10.1523/ENEURO.0370-20.2020 (PMC7877472; doi:10.1523/ENEURO.0370-20.2020)

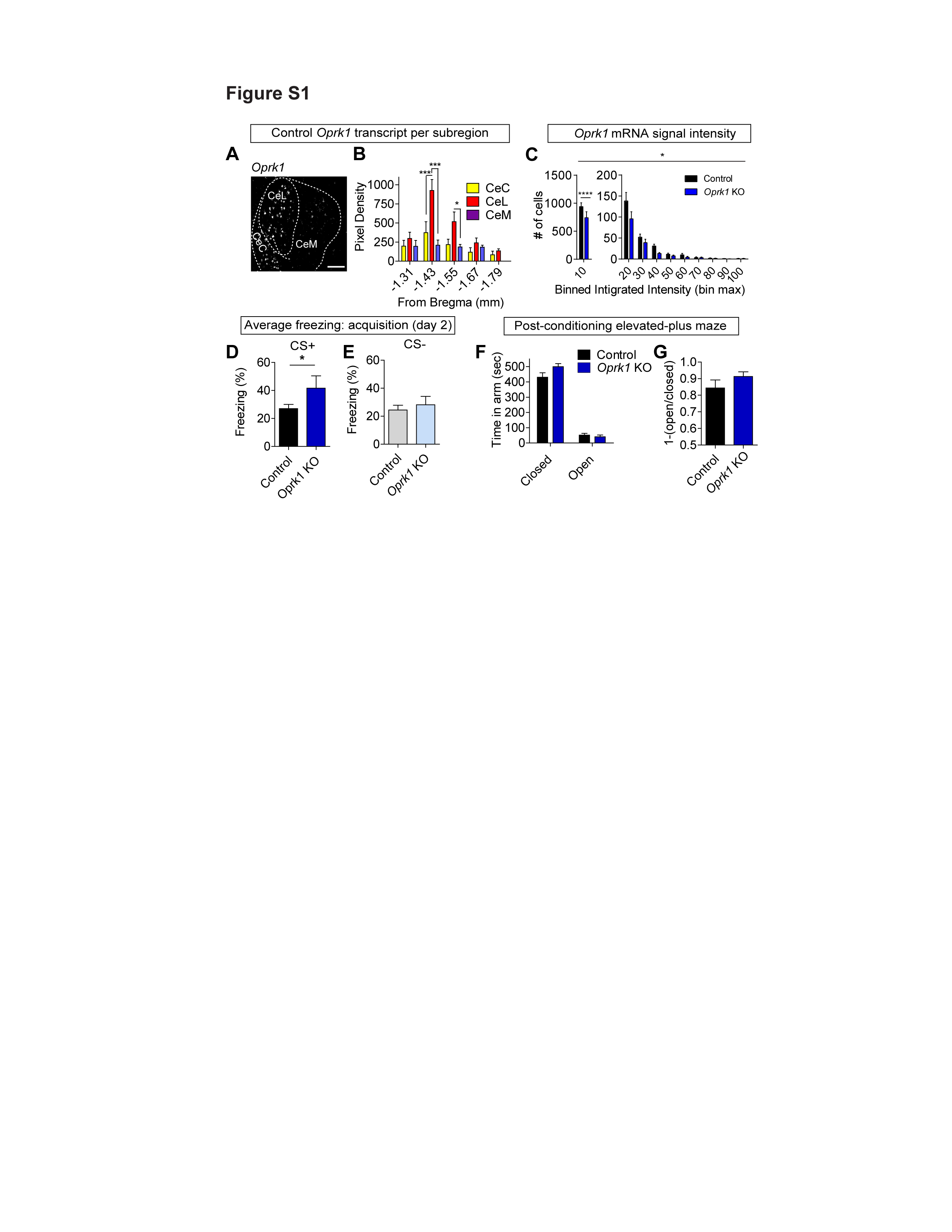

Supplement: Extended Data Figure 1-1 — Oprk1 signal intensity in CeA subdivisions and post-threat conditioning EPM in Oprk1 KO mice. A, Representative image of Oprk1 mRNA in the CeA. Scale bar: 100 μm. B, Average pixel intensity of Oprk1 mRNA fluorescent in situ signal in subdivisions of the CeA along the rostral to caudal axis (two-way ANOVA, F(6,27) = 3.87, p < 0.01, followed by Bonferroni’s multiple comparisons, *p < 0.05, ***p < 0.001). C, Binned Oprk1 transcript per cell in controls and Oprk1 KOs (two-way ANOVA, F(9,63) = 2.541, *p = 0.0148). D, E, Average freezing response during 10 trials of day 2 in control and Oprk1 KO mice during CS+ (D, Student’s t test, p < 0.05) and CS– (E). F, Time spent in open and closed arms of the EPM in control and Oprk1 KO mice following threat conditioning (control, N = 13; Oprk1 KO, N = 7). G, [1-(open/closed arm time)] in control and Oprk1 KO mice following threat conditioning. Data are presented as mean ± SEM. Download Figure 1-1, TIF file. [file enu-eN-NWR-0370-20-s04.tif]

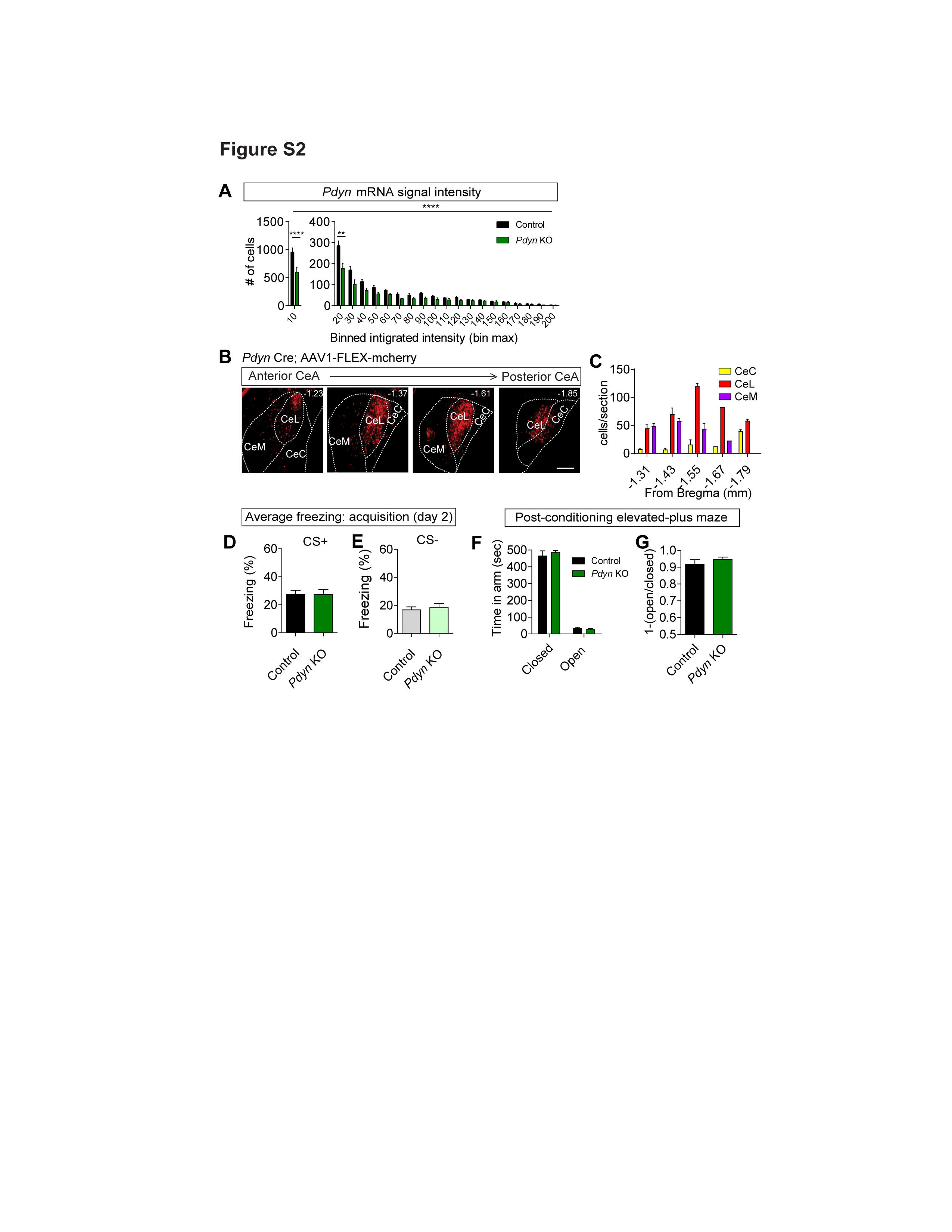

Supplement: Extended Data Figure 2-1 — Viral labeling of Pdyn-expressing cells in the CeA and post-threat conditioning EPM in Pdyn KO mice. A, Binned Pdyn transcript per cell in controls and Pdyn KOs (two-way ANOVA, F(19,114) = 9.011, ****p < 0.0001, followed by Bonferroni’s post hoc test, ****p < 0.0001, **p < 0.01). B, Representative images of mCherry expression in the CeA of PdynCre/+ mice. Scale bar: 100 μm. C, Quantification of mCherry-expressing cells in the CeA of PdynCre/+ mice. D, E, Average freezing response during 10 trials of day 2 in control and Pdyn KO mice during CS+ (D) and CS– (E). F, Time spent in open and closed arms of the EPM in control and Pdyn KO mice following threat conditioning (control, N = 9; Pdyn KO, N = 9). G, [1-(open/closed arm time)] in control and Pdyn KO mice following threat conditioning. Data are presented as mean ± SEM. Download Figure 2-1, TIF file. [file enu-eN-NWR-0370-20-s03.tif]

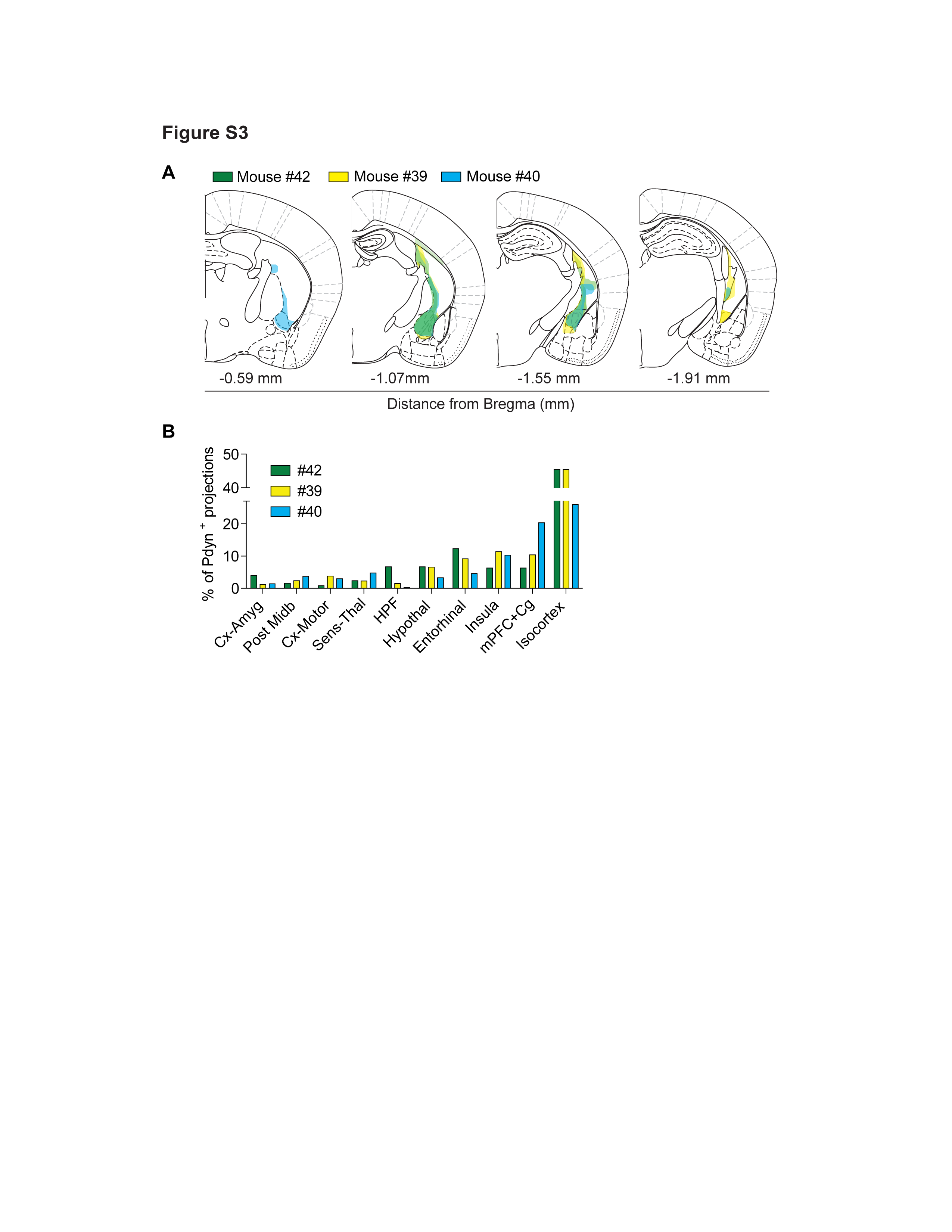

Supplement: Extended Data Figure 3-1 — Tissue distribution of Retro-AAV2-EGFP for mapping Pdyn-expressing inputs to the CeA. A, Cartoon images of mouse brain section along the rostral to caudal axis containing the CeA. Individual mice are pseudocolored to display viral spread at the injection site. B, Distribution of the percentage of EGFP-expressing neurons in individual mice in brain regions with Pdyn-expressing projections to the CeA. Cx-Amyg, cortex-amygdala transition zone; Post Midb, posterior midbrain; Cx-Motor, motor cortex; Sens-Thal, sensory thalamus; HPF, hippocampal formation; hypothal, hypothalamus; mPFC+Cg, medial prefrontal cortex and cingulated cortex. Download Figure 3-1, TIF file. [file enu-eN-NWR-0370-20-s02.tif]

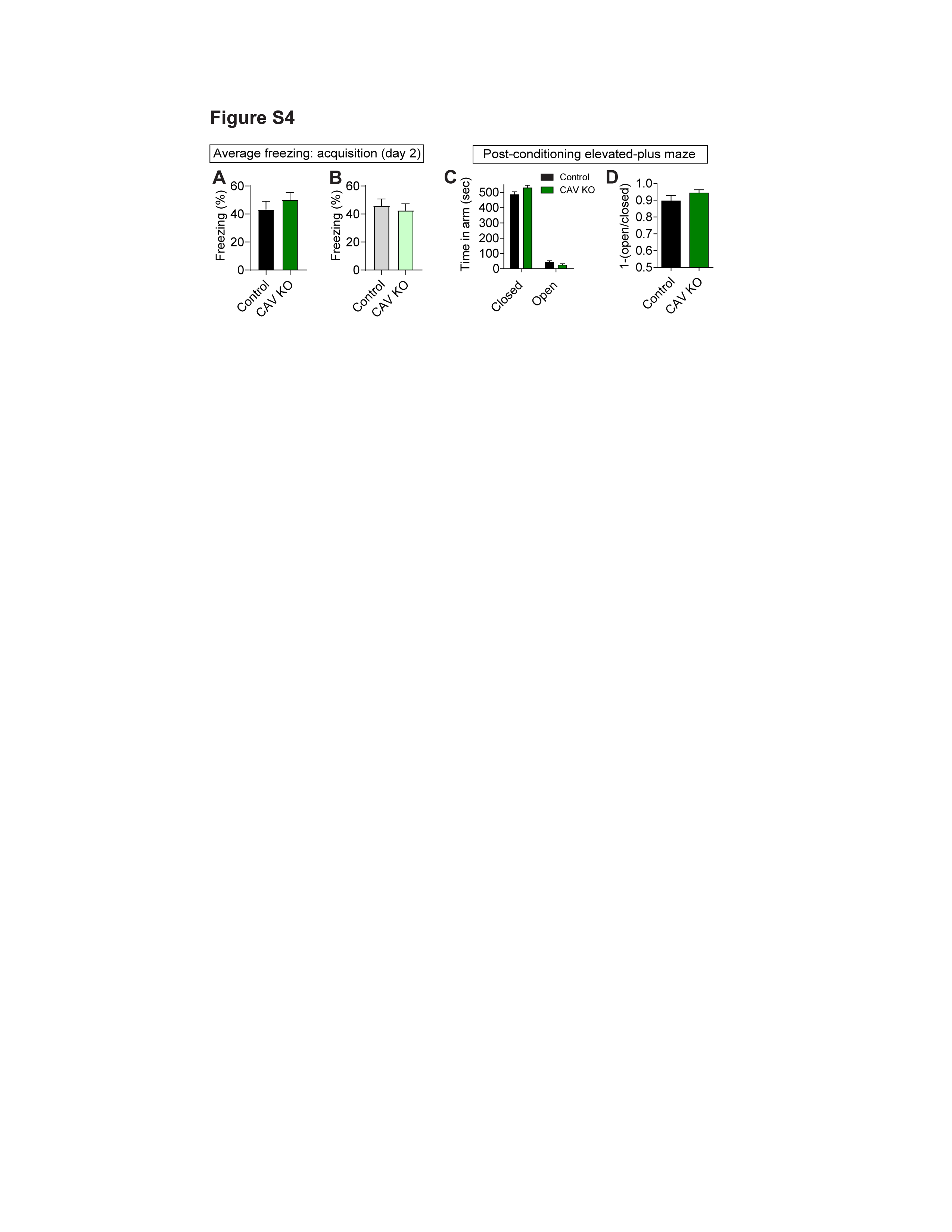

Supplement: Extended Data Figure 4-1 — Post-threat conditioning EPM in CAV KO mice. A, B, Average freezing response during 10 trials of day 2 in control and CAV KO KO mice during CS+ (A) and CS– (D). C, Time spent in open and closed arms of the EPM in control and CAV KO mice following threat conditioning (control, N = 15; CAV KO, N = 11). D, [1-(open/closed arm time)] in control and CAV KO mice following threat conditioning. Data are presented as mean ± SEM. Download Figure 4-1, TIF file. [file enu-eN-NWR-0370-20-s01.tif]
